# Supplementary material for: Curated and harmonised transcriptomics datasets of interstitial lung diseases
Source: Data Brief. 2025 Oct 14;63:112139. doi: 10.1016/j.dib.2025.112139 (PMC12581653; doi:10.1016/j.dib.2025.112139)
Supplement: Supplementary file 1 [file mmc1.zip › Supplementary_material/RNA-seq/fastQC reports/GSE124685/SRR8397214_fastqc.html]

SRR8397214.fastq.gz FastQC Report 

FastQC Report

Tue 30 Jan 2024  
SRR8397214.fastq.gz

## Summary

- Basic Statistics
- Per base sequence quality
- Per sequence quality scores
- Per base sequence content
- Per sequence GC content
- Per base N content
- Sequence Length Distribution
- Sequence Duplication Levels
- Overrepresented sequences
- Adapter Content

## Basic Statistics

| Measure | Value |
| --- | --- |
| Filename | SRR8397214.fastq.gz |
| File type | Conventional base calls |
| Encoding | Sanger / Illumina 1.9 |
| Total Sequences | 18823075 |
| Sequences flagged as poor quality | 0 |
| Sequence length | 8-354 |
| %GC | 53 |

## Per base sequence quality

## Per sequence quality scores

## Per base sequence content

## Per sequence GC content

## Per base N content

## Sequence Length Distribution

## Sequence Duplication Levels

## Overrepresented sequences

| Sequence | Count | Percentage | Possible Source |
| --- | --- | --- | --- |
| AAGCTCAGGGAGAGCCCTGTTAGGGCCGCCTCTGGCCCTAGTCTCAGACC | 364466 | 1.9362723678251295 | No Hit |
| GGGCCACGAGCTGAGTGCGTCCTGTCACTCCACTCCCATGTCCCTTGGGA | 311375 | 1.6542196213955478 | No Hit |
| GGGAATGTCTACGTGCGTATGCACGTGGCACTCTCTGCCCGAGGTCCGGG | 202652 | 1.0766147401527115 | No Hit |
| ACACTGAGGACTCTGTTCCTCCCCTTTCCGCCTAGGGGAAAGTCCCCGGA | 175796 | 0.9339387958662441 | No Hit |
| TCTCTGGGCTTCTATTTCGACCGCGATGATGTGGCTCTGGAAGGCGTGAG | 168493 | 0.8951406717552792 | No Hit |
| ACGCTCGTAGCCCTCGCGCTTCTCCTCGGCCAGTTCGCGGAAGAAGTGGC | 90828 | 0.48253539870610934 | No Hit |
| TGGTTCCTGGCAAGCCCATGTGTGTTGAGAGCTTCTCAGACTATCCACCT | 70449 | 0.3742693475959693 | No Hit |
| ACTTGATCCCAACTCATCTCTCATTTATTTCGGCTTCTTTTATTCCAGGA | 67286 | 0.35746550444069314 | No Hit |
| GCTGCTACAGGAGAATAGCAGACAGGTATAGTTAAGAAACAACTTTATTT | 67193 | 0.3569714300134277 | No Hit |
| TCTTTCTGGCCTGGAGGCTATCCAGCGTACTCCAAAGATTCAGGTTTACT | 61831 | 0.3284851173360357 | No Hit |
| AGCGGTTCCGCTGCCCTGAGGCACTCTTCCAGCCTTCCTTCCTGGGCATG | 55090 | 0.2926726903016643 | No Hit |
| TCACACTTCATGATGGAGTTGAAGGTAGTTTCGTGGATGCCACAGGACTC | 53874 | 0.28621253434946203 | No Hit |
| AGCTTAATGATGCTTTCTCTGGGCTTTTGGGGGAGGGTGTCCACCAGCTT | 53255 | 0.2829240174626091 | No Hit |
| CTAAGTGCTGTGTTGTCGTTCCCCCTGCTTAAAATAAAGTTGTTTCTTAA | 52431 | 0.278546411784472 | No Hit |
| ACACCCACCGCAACTGTCTGTCTCATATCACGAACAGCAAAGCGACCCAA | 51615 | 0.27421130713233627 | No Hit |
| ATCAAGACATGAGGGAGGCAGGGGCTCAGCTGAAGAAGCTGGTGGACACC | 48246 | 0.2563130625575258 | No Hit |
| ACACTGAGGACTCTGTTCCTCCCCTTTCCGCCTAGGGAAAGTCCCCGGAC | 45657 | 0.24255866801784512 | No Hit |
| TTCATGGTCTCGTCCATCAGCGCCCTCAGTTCCTGGGTGACCTGGGAGCT | 44925 | 0.23866982413872337 | No Hit |
| CGCTGGGTGCAGACACTGTCTGAGCAGGTGCAGGAGGAGCTGCTCAGCTC | 37278 | 0.19804415590970126 | No Hit |
| TCAGCACGGGCTCGTACTCTGCCACAAACTGATCACACTGCTTCTGGTAA | 36353 | 0.19312997477829738 | No Hit |
| ACATAGCAATTCAGGAAATTTGACTTTCCATTCTCTGCTGGATGACGTGA | 34086 | 0.18108624653517025 | No Hit |
| TGTTCCCTCTCCTCATGAGATTGGTGAAGAAAGTATTTGGCAAAGTTCTT | 33896 | 0.18007684716763867 | No Hit |
| CCACCTTCTCCATCGGCTCCACTGGCCTCGTGGTGTATGACTACCAGCAG | 33210 | 0.17643238418802454 | No Hit |
| GGGTACTTCAGGGTCAGGATGCCACGCTTGCTCTGGGCCTCGTCGCCCAC | 32409 | 0.1721769689596413 | No Hit |
| TCTGTCTTCTTCAGTTTCGACTTATCGAATTTCTCGATCTCAGCCATATC | 31608 | 0.16792155373125806 | No Hit |
| AAAGCCAAGAAAACCAACGATGCCAGATACTGAATTTCTATAGCACTGAC | 30276 | 0.16084513290203648 | No Hit |
| TTTACACCTGCTAGGGTGTTCAAAGGTCAGTGCTATAGAAATTCAGTATC | 29793 | 0.15827913345720612 | No Hit |
| CCGCAGACGCCGCCGCGATGCGCTACGTCGCCTCCTACCTGCTGGCTGCC | 27428 | 0.1457147676455627 | No Hit |
| TGTCCATGTCTTACTACTTTGACCGCGATGATGTGGCTTTGAAGAACTTT | 27218 | 0.14459911571302775 | No Hit |
| TCGGGCGCCCCAGACACCAGGCGTCATGGTGGGCATGGGCCAGAAGGACT | 26605 | 0.14134247459567578 | No Hit |
| CCCAGGGCAGAGGCTGACGACGCTGACTGGCAGGGCACCGACACAGTGTC | 26477 | 0.1406624581796545 | No Hit |
| TTGGCTTTCTCTTTCCTCTTCTCCTCCAGGGTGGCTGTCACTGCCTGGTA | 26453 | 0.14053495510165048 | No Hit |
| ATGATGTAGCAGCAGGTGCCAGGGGCTGGCTTGTAGGCGATCAGCAGCTG | 25841 | 0.1372836266125487 | No Hit |
| AATAAAAGCGAAAAGAAATGAAAATGTTACACTACATTAATCCTGGAATA | 24422 | 0.12974500712556264 | No Hit |
| CGGCATAAGTTGACACATGGTCCGCCTTGATGGCCCCAGCTCCTCGGAGA | 24338 | 0.12929874635254868 | No Hit |
| ACAGTGTATCTAGCTTTGGAAAACACAGGGGTCTGCCCTGTGAGCTGCTC | 24281 | 0.12899592654228917 | No Hit |
| AAAAGTGATGGACTGCCCATTGCTGGAGAAGACCTTCTCTCCTACTGTCA | 23819 | 0.12654149229071232 | No Hit |
| AAAGAAGAACATGTGATCATCCAGGCCGAGTTCTATCTGAATCCTGACCA | 23763 | 0.12624398510870302 | No Hit |
| AACATCTGTGTTGGGGAGAGTGGAGACAGACTGACGCGAGCAGCCAAGGT | 23709 | 0.12595710318319403 | No Hit |
| AGCACCAAGCAGGAGATCCTGGCTGCTCTTGAGAAAGGCTGCAGCTTCCT | 23383 | 0.12422518637363979 | No Hit |
| TCACTGATCAGACACACTAGTGTGGCCTTGTTGGCTTGGAGCTCCTCAGA | 23203 | 0.12326891328860985 | No Hit |
| CAGCTGGAGAAGGACTTCAGCAGCATGAAGAAGTACTGCCAAGTCATCCG | 22672 | 0.12044790768777151 | No Hit |
| GGCCTTCTTCTGGCGCAGAGGAAGCAGGCGCATCTGGGTGTGGGCAATGA | 21972 | 0.11672906791265508 | No Hit |
| AGCGGTTCCGGTGTCCGGAGGCGCTGTTCCAGCCTTCCTTCCTGGGTATG | 21409 | 0.11373805820781141 | No Hit |
| TACTCGTGCGCCTCGCTTTGCTTTTCCTCCGCAACCATGTCTGACAAACC | 20603 | 0.10945607983817734 | No Hit |
| TCAGAGCTGTGATCTTGAGAGCCCTCTCCTTGGCTTTCCTGCTGAGTCTC | 20073 | 0.10664038686558919 | No Hit |
| ACACTGAGGACTCTGTTCCTCCCCCTTTCCGCCTAGGGGAAAGTCCCCGG | 20068 | 0.10661382372433835 | No Hit |
| GGTTATAATCATTGGCAATGTTGTAATCAACAACCCATGTTATTTCTACA | 19746 | 0.10490315742778479 | No Hit |
| TTCTCCTCAGCTGGAGCAGCAGCAGTGGAGGGGGCAGGACCTCCTGCTGG | 19671 | 0.10450471030902231 | No Hit |
| TGAGTTGGCCAGAACTCTGAAAAGATTGGGAATGGATGGCTACAGGGGAA | 19655 | 0.10441970825701966 | No Hit |
| CGGTTGTACTTGCGGATGTAGTGCAGATAGTCTCGGCGGATGACAATGGT | 19503 | 0.10361218876299436 | No Hit |
| TTTGACATCCACTATGGCTCGGGCAGCCTCTCCGGGTACCTGAGCCAGGA | 19303 | 0.10254966311296108 | No Hit |
| GGGCCACGAGCTGAGTGCGTCCTGTCACTCCACTCCCCATGTCCCTTGGG | 19151 | 0.1017421436189358 | No Hit |
| GAAAAGTTTGGAAGAGGCAGAGAAATCCTGCTCTCCTCGCCTTCCAGCAG | 19038 | 0.10114181662666699 | No Hit |
| ATCAAATCCTGCAGACAAGGGGAGCCCTCAGTCTGCAGGGCTCCATAATG | 18902 | 0.10041929918464439 | No Hit |

## Adapter Content

Produced by FastQC (version 0.11.7)
